# Vector Development Timeline for Mucosal Vaccination and Treatment of Disease Using *Lactococcus lactis* and Design Approaches of Next Generation Food Grade Plasmids

**DOI:** 10.3389/fmicb.2018.01805

**Published:** 2018-08-14

**Authors:** Camila Prosperi de Castro, Mariana M. Drumond, Viviane L. Batista, Amanda Nunes, Pamela Mancha-Agresti, Vasco Azevedo

**Affiliations:** ^1^Laboratório de Genética Celular e Molecular, Departamento de Biologia Geral, Instituto de Ciências Biológicas, Universidade Federal de Minas Gerais, Belo Horizonte, Brazil; ^2^Kroton Educacional, Faculdade Pitágoras, Contagem, Brazil; ^3^Centro Federal de Educação Tecnológica de Minas Gerais, Coordenação de Ciências, Belo Horizonte, Brazil

**Keywords:** lactic acid bacteria, recombinant *L. lactis*, expression systems, DNA vaccine, food grade vectors

## Abstract

*Lactococcus lactis* has been used historically in fermentation and food preservation processes as it is considered safe for human consumption (GRAS—*Generally Recognized As Safe*). Nowadays, in addition to its wide use in the food industry, *L. lactis* has been used as a bioreactor for the production of molecules of medical interest, as well as vectors for DNA delivery. These applications are possible due to the development of promising genetic tools over the past few decades, such as gene expression, protein targeting systems, and vaccine plasmids. Thus, this review presents some of these genetic tools and their evolution, which allow us to envision new biotechnological and therapeutic uses of *L. lactis*. Constitutive and inductive expression systems will be discussed, many of which have been used successfully for heterologous production of different proteins, tested on animal models. In addition, advances in the construction of new plasmids to be used as potential DNA vaccines, delivered by this microorganism, will also be viewed. Finally, we will focus on the scene of gene expression systems known as “food-grade systems” based on inducing compounds and safe selection markers, which eliminate the need for the use of compounds harmful to humans or animal health and potential future prospects for their applications.

## *Lactococcus lactis*: the Model Lactic Acid Bacteria

Lactic acid bacteria (LAB) constitute a diverse group of Gram-positive microorganisms, including species of genera *Lactobacillus*, *Lactococcus*, *Streptococcus*, *Pediococcus* *Leuconostoc*, and *Oenococcus*, which, among other shared characteristics, have the capacity to convert sugars into lactic acid (Makarova and Koonin, 2007). Because they have long been used in fermentation and food preservation processes, most of these bacteria are considered safe for human consumption, possessing the GRAS (*Generally Recognized As Safe*) status (van de Guchte et al., 2006). Among the representatives of this group, *L. lactis* is the best characterized species and figures as the model organism for its study. This, in turn, is due not only to its evident economic importance, but also to the fact that this bacterium is a microorganism of easy manipulation and has its genome sequenced. A great number of genetic tools have been developed. Thus, *L. lactis* has also been widely used in the field of biotechnology for the production and delivery of antigens and cytokines to mucosal surfaces (Wells, 2011), and more recently as a vehicle for the delivery of DNA vaccines (Pereira et al., 2014, 2017; Pontes et al., 2014; Zurita-Turk et al., 2014; Souza et al., 2016; Mancha-Agresti et al., 2017).

This microorganism has several characteristics that make it an interesting vector for the production and delivery of biomolecules, especially for oral route, such as resisting stomach acidic environment and being able to survive in the gastrointestinal tract. Another attractive property of *L. lactis* is that it lacks lipopolysaccharides in its cell wall, which eliminates the risk of endotoxin shock. Finally, this bacterium is poorly immunogenic, and for this reason, it can be continuously used in immunization programs (Mercenier et al., 2000). Thus, *L. lactis* offers great versatility, and several vectors to be used in this microorganism have already been constructed, allowing a wide range of biotechnological applications, besides its use in the food industry.

## *Lactococcus lactis* as a Cell Factory—Proteins of Biotechnological Interest

The use of *L. lactis* as a cell factory for high-level protein production was developed using constitutive and inducible promoters. The history regarding the use of vectors for cloning exogenous genes in the LAB started with two cryptic broad host-range plasmids: pWV01 (Kok et al., 1984) and pSH71 (de Vos et al., 1997). The replicons of these plasmids have been the base for creating useful cloning vectors being able to replicate in a broad range of genera, such as *L. lactis* and also in *Escherichia coli* (recA+strain) (De Vos and Simons, 1994). In general, these small plasmids undergo a rolling-circle replication with chloramphenicol resistance. Another family of vectors derived from pAMbeta1 (from *Enterococcus faecalis*), which can only replicate in Gram-positive host strains, was constructed, pIL252 and pIL253, showing low and high copy plasmids, respectively, as having erythromycin resistance (Simon and Chopin, 1988; Kleerebezem et al., 1997b). These large plasmids replicate via “theta” replication and are equipped with *E. coli* replicons, generating pOri23 (Que et al., 2000), allowing efficient shuttling between Gram-positive and Gram-negative bacteria (O’Sullivan and Klaenhammer, 1993).

Afterward, several vectors containing constitutive or inducible promoters were developed, and they represent the basis of all expression systems in *L. lactis* and other LAB (Pontes et al., 2011). Although the high production of heterologous proteins in *L. lactis* has been achieved using constitutive promoters (De Vos, 1999), this continuous production of high-level protein could generate intracellular accumulation, which could lead to degradation in the cytoplasm and sometimes could be toxic to the cell. This drawback, together with the fact that inducible promoters offer better handling, makes them the vectors of choice. In 1995, the most promising and frequently used system based on genes involved in biosynthesis and regulation of nisin, an antimicrobial peptide whose biosynthesis is encoded by a cluster of 11 genes, was developed (Kuipers et al., 1995). The NICE system (nisin-controlled expression), as it is called, comprises NIS operon regulatory elements, NisR and NisK code, for the regulator two-component system. The histidine-protein kinase NisK located in the cytoplasmic membrane binds the nisin molecule and activates NisR via phosphorylation, which in turn induces transcription of two promoters in the nisin gene cluster: PnisA and PnisF. In fact, this system has been demonstrated to have high protein production and is also easy to use as it has been extensively used in the industry. However, nisin has a high cost limiting its use. Due to nisin, researchers developed another system that is inducible by xylose sugar (Miyoshi et al., 2004). The xylose-inducible expression system (XIES) has the PxylT promoter, and in the presence of other sugars such as glucose, fructose, and/or mannose, it is tightly repressed. Thus, in the presence of xylose, PxylT is transcriptionally activated (Lokman et al., 1994). Another important characteristic of this system is its capacity to produce cytoplasmic or secreted proteins, and it can be easily switched on or off by adding either xylose or glucose, respectively (Miyoshi et al., 2004). As a sugar is used to operate this system, it is considered cheap and very useful for implementation in biotechnological processes. The secretion of heterologous proteins has advantages over intracellular-expressed ones, in the way in which only a simple purification step is necessary, higher yields are reached, and better target interactions are achieved (Le Loir et al., 2005).

Two other inducible systems for *L. lactis* were described recently. One is the zinc-inducible expression system, called ZIREX. This system allows induction of genes regulated by zinc ions and the pneumococcal repressor SczA (Mu et al., 2013). The other inducible system for *L. lactis* is stress-inducible controlled expression system (SICE). This system uses a promoter of the *L. lactis* groESL operon, whose expression is induced under stress conditions, which does not need exogenous induction, nor the presence of regulatory genes (Benbouziane et al., 2013). Characteristics of all these vectors are summarized in **Table 1**. Many of these vector applications have been described with satisfactory results. A recent report showed the gastrointestinal passage time of bacteria and their spatial localization in the gut. To this end, the authors used a plasmid from the NICE system (pNZ8148) in which IRFP reporters (pNZIRFP713 and pNZ-IRFP682) were cloned, resulting in near-infrared fluorescent proteins being expressed in the LAB (Berlec et al., 2015).

**Table 1:** Characteristics of constructed *L. lactis* vectors for heterologous protein production.

Plasmid/ System	Characteristics	Cloned genes	Reference
pWV01	Lactococcal cryptic plasmid, rolling circle replication, (RCR) type, Cm^r^, 2.1 kb size, P23 promoter.	–	Kok et al., 1984
pSH71	Rolling circle replication (RCR) type, Cm^r^.	–	de Vos et al., 1997
pIL252	Low-copy-number, broad-host- range cloning vector, theta-replication, Ery^r^, 4.7 kb size, P23/59 promoters.	–	Simon and Chopin, 1988
pIL253	High-copy-number, broad-host-range cloning vector, theta-replication, Ery^r^, 5.2 kb size P23/P32 promoters.	–	Kleerebezem et al., 1997a
pORI23	High-copy-number, broad-host-range cloning vector, theta-replication, *oriCol*E1 origin, Ery^r^, 5.2 kb size P23 promoter	*Luciferase*	Que et al., 2000
NICE system	Nisin controlled gene expression system, Cm^r^, PnisA and PnisF. promoters. Secreted heterologous protein *L. lactis* NZ900 is the only host strain (NisK, nisR in genome). Nisin induced.	*GroEL* *Catalase* *HPV-16 E7/IL-12* *Human -Leptin*	Kuipers et al., 1995 Miyoshi et al., 2006 de Moreno de LeBlanc et al., 2008 Bermudez-Humaran et al., 2005 Bermúdez-Humarán et al., 2007
XIES system	Xylose inducible expression system. Cm^r^, pXylT promoter. Two versions of heterologous proteins, either cytoplasmic or secreted. Xylose induced.	*Mirabilis MrpA* *fimbrial protein* *IL10* *15-Lox-1* *hsp65*	Miyoshi et al., 2004 Scavone et al., 2007 Marinho et al., 2010 Saraiva et al., 2015; Carvalho et al., 2016 de Azevedo et al., 2012
ZIREX system	Zinc-inducible expression system. Cm^r^ pczcD promoter with SczA gene (for its regulatory protein).	*gfp*	Mu et al., 2013
SICE System	Stress Inducible Controlled Expression system. Cm^r^, groESL promoter, Stress conditions induced.	*IL10* *HPV-16E7*	Benbouziane et al., 2013

*Em^r^-erythromycin resistance gene, Cm^r^ - Chloramphenicol resistance gene, hsp65: heat-shock protein coding sequence of Mycobacterium leprae, GroEL: heat-shock protein coding sequence of B. abortus, 15-Lox-1: human 15 lypoxygenase coding sequence, HPV-16/E7: human papillomavirus type 16 cell wall-anchored E7 Antigen.*

Concerning the XIES system, some therapeutic molecules such as 15-lipoxygenase were cloned in it. *L. lactis* (pXIES:CYT:*15lox-1*)-fermented milk was effective in the prevention of intestinal damage associated with inflammatory bowel disease (IBD) in a trinitrobenzenesulfonic acid-induced IBD mouse model (Saraiva et al., 2015) and, in addition, a decrease in IFN-γ and IL-4 was also observed. Also, an increase in IL-10 was observed in a dextran sodium sulfate-induced (DSS-induced) IBD mouse model, where mice were orally administrated with the culture of this strain (Carvalho et al., 2016). *L. lactis* (pXIES:*hsp65*) expressing the 65-kDa heat shock protein from *Mycobacterium leprae* (de Azevedo et al., 2012) was able to prevent the development of experimental autoimmune encephalomyelitis in C57BL/6 mice which received this bacterium by oral administration. The reduction of IL-17 and the increase of IL-10 in mesenteric lymph node and spleen cell cultures were observed (Rezende et al., 2013). The same recombinant strain was tested in the DSS-induced colitis mouse model. In colonic tissue levels, proinflammatory cytokines (IFN-γ, IL-6, and TNF-α) were reduced by increasing IL-10 production. An expansion of regulatory T cells (CD4^+^Foxp3^+^ and CD4^+^LAP^+^) was also observed in the spleen, as well as in mesenteric lymph nodes. Thus, these data indicate that oral pretreatment with genetically modified *L. lactis* Hsp65 protein production is able to prevent DSS-induced colitis in C57BL/6 mice (Gomes-Santos et al., 2017).

## Dna Vaccine Vectors

Since the early 1990s, several studies have been conducted to test the efficiency of DNA vaccines in the prevention or treatment of different diseases (Huang and Gorman, 1990; Galvin et al., 2000). Modern vaccinology employs plasmids obtained from bacteria that encode antigen polypeptide sequences. These constructions are made using classical molecular biology techniques and more recently molecular techniques such as synthetic biology. There are many advantages of using bacterial plasmids as a vaccine strategy, and some of them are related to their stability at room temperature and reduction of production costs once the purification of the protein in interest is not required, which increases safety in administration (Suschak et al., 2017). Since these vaccine techniques began to be developed, many studies have been conducted aiming at empowering the delivery of these plasmids to host cells, and among these different strategies, we can cite, for example, the different routes of administration and use of adjuvants. However, much emphasis has been placed on the design of vectors that will be used as a vaccine platform (Sørensen et al., 2005; Oliveira and Mairhofer, 2013; Williams, 2014; Suschak et al., 2017).

Currently, there are very few groups worldwide working on the development of vectors for DNA vaccines using *L. lactis* as delivery vehicles, and in recent years, only a few papers have been published in the scientific literature (Guimarães et al., 2009; Tao et al., 2011; Mancha-Agresti et al., 2016; Yagnik et al., 2016, 2017). Vectors generally exhibit some common features such as the presence of a strong eukaryotic promoter, such as cytomegalovirus, multiple cloning sites, a polyadenylation signal in addition to a prokaryotic region that includes a selection marker, usually an antibiotic resistance gene, as well as a prokaryotic origin of replication (Kutzler and Weiner, 2008). In 2009, our research group published a new vector for DNA delivery, pValac (vaccination using lactic acid bacteria), using lactococci. This vector exhibits the characteristics cited above including bovine growth hormone (BGH polyA) polyadenylation sequence, prokaryotic region that allows its replication in both *E. coli* and *L. lactis* strains, and chloramphenicol resistance gene as a selection marker, in addition to presenting a rolling circle origin of replication, which contributes to the relatively small size of the plasmid (3742 bp). *L. lactis* carrying pValac was able to efficiently deliver the vector to eukaryotic cells, allowing expression of green fluorescent protein (GFP) that could be observed by fluorescence microscopy and flow cytometry (Guimarães et al., 2009). Tao et al. (2011) published a paper describing the construction of the pLKV1 vector (4400 bp), which is quite similar to pValac. However, its selection marker is the kanamycin resistance gene, which is seen as an advantage due to the Food and Drug Administration recommending its use in DNA vaccines. The DsRed2 gene, which encodes a red fluorescent protein, was cloned in it. The functionality of this plasmid was verified by visualizing the expression of this protein in epifluorescent microscopy 48 h after the transfection of Caco-2 cells (Tao et al., 2011). However, there are no reports in the literature about *in vivo* studies using this plasmid. More recently, Yagnik et al. (2016) published the construction of the pPERDBY vector (4800 bp), which, in addition to sharing features common to other vectors already mentioned above, shows the polyadenylation signal of SV40 and the enhanced green fluorescent protein, eGFP, linked to the multiple cloning sites. This feature was designed to facilitate other protein expression, since the eGFP is expressed fused to the protein of interest where the presence of immunostimulatory CpG motifs, which could act as adjuvants, has been described in it. In the Chinese hamster ovary cells (CHO) cell transfection assay, it was possible to observe eGFP expression, demonstrating the functionality of the vector. However, in the flow cytometry analysis, the percentage of cells expressing the reporter protein was extremely low, showing only 0.53% of positive cells (Yagnik et al., 2016). In 2016, our research group published a study relating to the construction of the pExu vector (extra chromosomal unit) with 6854 bp, which in comparison with the described vectors presents the “theta” type origin of replication, which gives it both greater structural and segregational stability. Eukaryotic cells were transfected with pExu:*egfp* plasmid and analyzed by confocal microscopy and flow cytometry 48 h after transfection. The flow cytometric analysis showed that 18.7% cells were positive for eGFP expression. In addition, *in vivo* expression kinetics were performed on mice that had received the *L. lactis* (pExu:*egfp*) strain by oral administration. The results demonstrated that intestinal cells, specifically in the duodenal region of the bowel, were able to produce the protein of interest from 12 h to 72 h after treatment (Mancha-Agresti et al., 2016) in high amounts. These interesting results would be beneficial from a therapeutic standpoint because it reduces the need for multiple doses in a short period of time. **Table 2** summarizes some characteristics of major plasmids constructed for DNA vaccine.

**Table 2:** Main characteristics of vectors constructed for DNA vaccine uses.

Plasmid	Characteristics	Cloned genes	Reference
pValac	Eukaryotic expression vector (pCMV/RCR/RepA/RepC/Cm^r^/BGH polyA). Size: 3742 pb	*gfp* *Ag85A* *ESAT-6* *IL-10* *IL-4*	Guimarães et al., 2009 Mancha-Agresti et al., 2017 Pereira et al., 2014 Zurita-Turk et al., 2014 Souza et al., 2016
pLKV1	Eukaryotic expression vector, (pCMV/RCR/RepA/RepC/Km^r^/BGH polyA). Size: 4400 pb	*DsRed2* *GFPmut3*	Tao et al., 2011
pPERDBY	Eukaryotic expression vector (pCMV/RCR/RepA/RepC/Cm^r^/BGH SV40 polyA/ *eGFP*). Size: 4800 pb	*egfp*	Yagnik et al., 2016
pExu	Eukaryotic expression vector (pCMV/TER/RepD/RepE/Ery^r^/BGH polyA). Size: 6854 pb	*egfp* *mCherry* *hsp65*	Mancha-Agresti et al., 2016 (Personal communication Dr. Azevedo) (Personal communication Dr. Azevedo)

*pCMV cytomegalovirus promoter; RepA, RepC, RepD, and RepE replication origins; Cm^r^-chloramphenicol resistance gene; Ery^r^-erythromycin resistance gene; Km^r^-kanamycin resistance gene, egfp enhanced green fluorescent protein coding sequence; RCR: a rolling circle origin of replication, TER: a theta origin of replication. Ag85A coding sequence of Mycobacterium tuberculosis, ESAT-6: early secreted antigenic target coding sequence of Mycobacterium tuberculosis, DSRed2: red fluorescent protein coding sequence, GFPmut3: mutant green fluorescent protein coding sequence, egfp; enhanced green fluorescent protein coding sequence, mCherry: red fluorescent protein coding sequence, hsp65: heat-shock protein coding sequence of Mycobacterium leprae.*

## Food-Grade Vectors for *Lactococcus lactis*

The use of *L. lactis* for the production of heterologous proteins, as well as for the delivery of DNA vaccines, is currently a promising reality. However, for further clinical trials, the assessment and minimization of risks to human health will be necessary. One of the major concerns related to the use of recombinant bacteria in clinical trials is related to the safety of their use by the host. Historically, cloning and expression vectors are designed using antibiotic resistance genes as marker selection; therefore, this feature may make it unfeasible to use extra-laboratories of *L. lactis* in the food and pharmaceutical industry.

Many questions are raised, not only about this specific issue, but also about the long-term adverse effects, as well as questions about possible integration into genome, dissemination and toxicity of these plasmids during human trials. To minimize these concerns, the development of new expression systems composed of safer elements, called food-grade systems, that allow selection, induction, and maintenance in the host is necessary to overcome the use of conventional gene expression systems and preserve the GRAS status of this bacterium (Cotter et al., 2003; Landete, 2017). Thus, some tools can be used, such as optimization of vector design, allowing the construction of food grade plasmids, without antibiotic resistance markers, for example, which could increase the safety in use. In addition, bioinformatics tools can aid in this process, allowing the construction of more robust vectors without, however, compromising the safety in the use to treat diseases in humans (Cho et al., 2018).

Among the main mechanisms of resistance and induction used for the development of food-grade vectors such as auxotrophic complementation, chromosome integration, use of sugars, resistance to heavy metals, and more recently, the use of CRISPR/Cas technology can be mentioned (Berlec and Strukelj, 2009; Landete, 2017; Berlec et al., 2018). As an example of auxotrophic complementation, MacCormick and coworkers developed a food-grade system based on lactose metabolism of the *L. lactis* MG5267 strain. This strain, which has *lac* operon in its chromosome, was subjected to deletion of the *lacF* gene, and when this gene was expressed in a plasmid, its ability to metabolize lactose was restored, presenting potential for industrial use (MacCormick et al., 1995). Another selection system based on threonine complementation was described. For this reason, researchers constructed the pJAG5 vector containing the gene that encodes homoserine dehydrogenase-homoserine kinase protein as a selective marker, which catalyzes two steps of the conversion of the aspartate into threonine. This vector, when used in an *L. lactis* MG1363 strain that presents deletions in two genes encoding threonine biosynthetic enzymes, proved to be quite stable and was able to express green fluorescent protein in eukaryotic cells (Glenting et al., 2002). In another study, a purine auxotrophic *L. lactis* DN209 strain (Dickely et al., 1995) was transformed with replicative food-grade plasmid pFG1 coding for genes producing peptidases from *Lactobacillus helveticus*. This strain was able to reduce the ripening period in cheese manufacturing, as well as producing special varieties of cheese (Joutsjoki et al., 2002). Besides auxotrophic complementation, resistance to heavy metals can also be explored with the aim of developing food-grade expression systems for *L. lactis* (Landete, 2017). The pND302 vector identified in *L. lactis* M71 strain, for example, offers resistance to cadmium (Liu et al., 1996). Even though it seems paradoxical, the use of these markers is safe, because this vector uses a theta origin of replication, thus being stable even in the absence of selective pressure (Emond et al., 2001).

However, it is important to note that some food-grade vectors present structural instability, and to circumvent this problem, some systems based on chromosomal integration have been developed (Wegmann et al., 1999; Gosalbes et al., 2000; Sheng et al., 2015). Perhaps the most promising example of this technique is the work of Steidler et al. (2003). These researchers replaced thymidylate synthase gene (thyA) of *L. lactis* with a synthetic human IL10 gene by double homologous crossover (Steidler et al., 2003). This strain contains no antibiotic resistance markers, and because of its thymidine auxotrophy, it cannot spread in the environment, making it one of the safest genetically modified organisms ever designed. The efficacy of this strain was assessed in a first phase 1 clinical trial in a patient with Crohn’s disease, and the results from this study revealed that the use of genetically modified *L. lactis* to deliver therapeutic molecules, such as IL-10, at the mucosal level is a viable strategy in humans with chronic intestinal inflammation (Braat et al., 2006). Another elegant report showed a recombinant strain of LAB, capable to both produce and secrete the mucosal protectant human trefoil factor 1 at the site of the targeted oral mucosa [*L. lactis* (AGO13)]. This strain was engineered based on the same strategy (deficient in the gene encoding thymidylate synthase), being a food-grade strain. Results in patients with oral mucositis (phase 1 clinical trial) demonstrated that this strain was able to ameliorate the symptoms of this disease (Limaye et al., 2013).

Although research works with recombinant LAB show promising results in animal models of human disease, these particular ones were the only studies in which recombinant bacteria were used in human clinical trials, demonstrating its potential as a biotechnological tool for the treatment of diseases, but still remaining as a proof of concept. Concerning the regulation of gene expression in food-grade vectors, sugar, temperature, and pH-induced systems are highlighted. The promoter P170 present in *L. lactis* is inducible by the accumulation of lactic acid at pH 6.0–6.5, when the culture is reaching the stationary phase (Madsen et al., 1999). Therefore, this self-inducible promoter could be considered for the construction of new food-grade expression systems.

P1 and P2 are classic examples of temperature-induced promoters. The repressor of the P2 promoter is inactivated when the medium temperature reaches 40°C. Thus, shifting growth temperature to 24–42°C results in an increase of gene expression controlled by this promoter (Sanders et al., 1998). Sugars can be a safe and inexpensive way to induce gene expression in food-grade systems. Payne and colleagues developed a food-grade expression system induced by lactose; they used the previously mentioned *L. lactis* strain MG5267, which possesses the lactose operon integrated in the bacterial chromosome. After the lysin gene from *Listeria monocytogenes* bacteriophage LM-4 was integrated into the chromosome, the chromosomal *lacG* gene, which encodes phospho-beta-galactosidase, was inactivated by a double cross-over event. Thus, the expression of the lysin gene was shown to be regulated by growth in the presence of lactose, proving to be an important strategy for controlled protein expression in *L. lactis* (Payne et al., 1996).

## Perspectives for Development of Next Generation Vectors

*Lactococcus lactis*, the model lactic acid bacteria, has enormous potential to be used as a biofactory for the production of numerous proteins of medical and industrial interests, as well as a carrier vehicle for DNA vaccines. Nowadays, this is a reality on the laboratory scale. As new information concerning this microorganism emerges, new possibilities for its use are being contemplated. Constant discoveries in the areas of genomics, transcriptomics, and proteomics of lactic acid bacteria provide us information for new approaches for the construction of the next generation of vectors. These should include the investigation of *L. lactis* cryptic plasmids, as well as exploration of new selection markers that exclude any type of resistance to antibiotics. In addition, features such as host range, stability, and copy number of plasmids must be considered and also the type of replication origin to be used in vector construction. The theta-type replication mechanism is indicated for reasons already mentioned in previous sections. Moreover, food-grade systems can also be developed by integrating vectors into the host’s chromosomal DNA allowing more stability. Finally, mechanisms of gene expression induction that include the use of cheap and non-harmful compounds such as sugar, pH, or temperature should be considered for future biomedical, biotechnological, and industrial applications. Furthermore, the most promising technique in the field of genetic studies is the CRISPR Cas9 (clustered regularly interspaced short palindromic repeats) system. CRISPR is known as the prokaryote adaptive immune system that provides resistance against foreign DNA, such as plasmids and bacteriophage (Bolotin et al., 2005; Deveau et al., 2008). This system has been studied for the last 30 years (Ishino et al., 1987), but only in 2013 the first experiments were conducted, demonstrating its functionality as an editing genome tool (Cong et al., 2013; Mali et al., 2013). This system has been used in different studies to help understand both disease models and therapeutic schemes for several diseases (Jiang et al., 2013; Makarova et al., 2015; Di Bella et al., 2016).

Although several studies have been carried out with bacterial species using this strategy for genomic editing, only few studies are reported to be using LAB (Oh and van Pijkeren, 2014; Sanozky-Dawes et al., 2015; Song et al., 2017; Berlec et al., 2018). In this way, this technique can be explored for the development of food-grade expression systems and opens perspectives for the use of *L. lactis* in clinical routine in the near future.

## Author Contributions

CP, MD, and PM-A conceptualized the study. CP, MD, PM-A, VB, and AN wrote the original draft of the paper. CP, MD, PM-A, and VA reviewed and edited the manuscript. VA acquired funding and supervised the study.

## Conflict of Interest Statement

The authors declare that the research was conducted in the absence of any commercial or financial relationships that could be construed as a potential conflict of interest.
